# Valorization of Camelina Cake by Fractionation: Characterization of Nutritional and Functional Properties

**DOI:** 10.3390/foods14193437

**Published:** 2025-10-08

**Authors:** Slađana Rakita, Nedeljka Spasevski, Strahinja Vidosavljević, Zorica Tomičić, Ivan M. Savić, Ivana M. Savić Gajić, Olivera Đuragić, Ana Marjanović Jeromela

**Affiliations:** 1 Institute of Food Technology, University of Novi Sad, Bulevar Cara Lazara 1, 21000 Novi Sad, Serbia; nedeljka.spasevski@fins.uns.ac.rs (N.S.); strahinja.vidosavljevic@fins.uns.ac.rs (S.V.); zorica.tomicic@fins.uns.ac.rs (Z.T.); olivera.djuragic@fins.uns.ac.rs (O.Đ.); 2 Faculty of Technology in Leskovac, University of Niš, Bulevar Oslobodjenja 124, 16000 Leskovac, Serbia; savicivan@tf.ni.ac.rs (I.M.S.); savicivana@tf.ni.ac.rs (I.M.S.G.); 3 Institute of Field and Vegetable Crops, Maksima Gorkog 30, 21000 Novi Sad, Serbia; ana.jeromela@ifvcns.ns.ac.rs

**Keywords:** oilseed cake valorization, protein source, tocopherols, antinutritional factors, omega-3 fatty acids, antioxidative potential, water absorption capacity

## Abstract

The objective of this study was to investigate the effects of fractionation by sieving on cold-pressed camelina cake by separating it into particle-sized fractions and evaluating their nutritional and functional properties. Two *Camelina sativa* varieties, NS Zlatka and NS Slatka, were mechanically cold-pressed using a screw press then ground into flour. The resulting material was fractionated into three particle-sized fractions, >250 µm, 250–180 µm, and <180 µm, using a laboratory dry sieving system. Both the whole cake and the separated fractions were analyzed for proximate composition, amino acid and fatty acid profiles, tocopherol content, antioxidant potential, color characteristics, and water and oil absorption capacities. The results indicated that the finest cake fraction (<180 µm) from both camelina varieties contained the highest content of protein (~40%), fat (17–19%), essential amino acids (~17 g/100 g), γ-tocopherols (254–266 mg/kg), and the lowest content of condensed tannins (0.5–0.9 g/kg). It also displayed a lighter color and increased yellowness. However, it contained the highest concentrations of glucosinolates (24–27 μmol/g) and phytic acid (38–41 g/kg). In contrast, the coarsest fraction (>250 µm) had increased crude fiber content (13–15%), higher antioxidant potential, the greatest water absorption capacity, and a darker color with a more pronounced reddish color. It also contained the lowest levels of glucosinolates (19–21 μmol/g) and phytic acid (17–20 g/kg). In conclusion, whole camelina cake and its fractions demonstrate considerable potential for use in animal feed and a variety of human nutritional products, due to their favorable nutritional composition and functional properties. Fine fractions with high levels of antinutritional compounds could be used as a substrate for the extraction of bioactive compounds and may find further application in the cosmetic and pharmaceutical industries.

## 1. Introduction

The development and adoption of new crops for food and feed is becoming increasingly important in light of the growing global population, the urgent need to meet rising nutritional demands, and the escalating challenges posed by climate change. As pressure mounts on agricultural systems to produce more with fewer resources, there is a strong push for sustainable crop species that use less water, fertilizers, and pesticides, emit fewer greenhouse gases, and are more resilient to environmental stresses [1,2]. Alternative protein crops, in particular, are gaining attention for their potential to enhance food security and reduce the ecological footprint of both human diets and animal production systems. A report by the European Commission has identified a range of such crops already present in Europe that could contribute to closing the continent’s protein gap and promoting renewable raw materials for feed and industrial use [3].

Among the emerging alternative crops, *Camelina sativa* (L.) Crantz has distinguished itself as an attractive candidate, drawing growing attention from researchers due to its remarkable agronomic and nutritional advantages. Owing to its valuable nutritional properties [4], camelina seed offers promising potential in animal feeding strategies aimed at enhancing the nutritional quality of animal-derived products for human consumption [5]. During the mechanical cold-pressing of camelina seeds for oil extraction, a considerable quantity of by-product is generated. This residual press cake is a nutritionally and biologically valuable material with significant potential for further utilization, particularly in animal feed or bio-based applications. Camelina cake is characterized by high protein content, exceeding that of the seed itself, with a favorable amino acid profile, as well as a considerable fat content rich in ALA. In addition, it contains significant amounts of bioactive compounds originating from the seed [6], including tocopherols, phenolic acids, and flavonoids [7]. Such a composition makes it an attractive candidate for inclusion in food systems; however, its application in human nutrition has been explored only to a limited extent. For example, Ernosh et al. [8] incorporated camelina cake at levels of up to 10% in bakery products, recommending its use in items such as hamburger and sandwich buns. In another study, Łopusiewicz et al. [9] utilized camelina seed cake as a raw material for producing a functional yogurt-like fermented beverage using yogurt starter cultures. In contrast, its use in animal feeding has been far more extensively investigated and adopted. Camelina cake is valued as a cost-effective source of energy, protein, and essential omega-3 and omega-6 fatty acids, with a market price of approximately USD 0.25/kg, lower than that of rapeseed cake [10], which further supports its attractiveness for use in animal feeding. Extensive research has confirmed its suitability in the diets of monogastric animals [11,12,13] and ruminants [14,15], demonstrating its potential to modulate the nutritional profile of animal-based products by increasing the content of beneficial omega-3 and omega-6 polyunsaturated fatty acids (PUFA). However, the use of camelina seed cake in food and feed is limited by the presence of antinutritional factors, including glucosinolates, erucic acid, tannins, and phytic acid. Strategies to mitigate these compounds include thermal processing, autoclaving, soaking, enzymatic treatments, and fermentation [16]. While thermal methods can decrease nutrient availability, enzymatic and fermentation processes, although effective, may significantly increase production costs. Pojić et al. [17] demonstrated the use of dry fractionation via sieving as a viable approach to concentrate both nutritional and antinutritional components in hempseed cake. Similarly, sieving has been applied to oilseed cakes such as flaxseed, rapeseed, hempseed, safflower, sunflower, pumpkin, milk thistle, and poppy to evaluate the distribution of nutritionally and biologically valuable constituents, as well as selected functional properties across particle size fractions [18]. In addition, canola meal has been fractionated using sieving techniques to explore the potential for the application of different particle sizes in animal and aquaculture feed [19]. Previous research has shown that the fractionation of camelina cake can affect the properties of biopolymer films derived from cake fractions [20]. However, to the best of our knowledge, no studies have reported on the application of sieving to camelina cake with the aim of concentrating desirable nutritive and bioactive compounds or reducing antinutritional compounds for potential applications in food and feed.

Therefore, the objective of this study was to investigate the effects of fractionation by sieving on cold-pressed camelina cake by separating it into particle-sized fractions and evaluating their nutritional and functional properties. It was hypothesized that sieving would yield fractions enriched in protein and bioactive compounds while simultaneously affecting antinutritional factors. Additionally, due to the naturally present mucilage that coats the seeds and remains in the cake after oil pressing, the question arises regarding the effect of fractionation on water- and oil-binding capacities, which opens up the possibility for the fortification of various types of food products. The findings of this study will serve as a roadmap for prospective use of the obtained cake fractions, based on their characteristics, in both food and feed systems.

## 2. Materials and Methods

### 2.1. Plant Material

**Figure 1:**
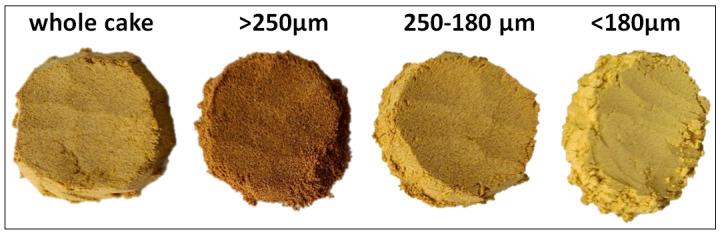
Whole camelina cake and its fractions obtained by sieving.

Two *Camelina sativa* varieties NS Zlatka and NS Slatka, developed by the Institute of Field and Vegetable Crops, Novi Sad, Serbia, were cultivated in 2024 in the South Bačka District, located in the northern part of Serbia. Cleaned and dried camelina seeds were mechanically pressed using a screw press (Ulimac Machine, Ankara, Turkey; 1.5 kW, capacity 5–45 kg/h) operated at a temperature below 50 °C. Then, the obtained camelina cake was ground into flour in a laboratory mill (Foss KN 295 Knifetec, Foss, Hillerød, Denmark) equipped with a water-cooling system to prevent additional heating during milling. The ground material was then fractionated into three particle-sized fractions, >250 µm, 250–180 µm, and <180 µm, using a laboratory dry sieve system (Bühler AG, Uzwil, Switzerland) (Figure 1). All analyses were performed on the whole cold-pressed camelina cake and individual fractions.

### 2.2. Methods

#### 2.2.1. Proximate Composition

Camelina seed cakes and fractions were analyzed for moisture content [21], crude protein [22], crude ash [23], and crude fat [24]. The crude fiber content was determined according to the Ankom method (AOCS Ba 6a-05) [25], using the Ankom 2000 Fiber Analyzer (Ankom Technology, Fairport, NY, USA). The glucosinolate content was determined by the MSZ-08-1908 method [26]. The content of condensed tannins was analyzed according to Ilić et al. [4], while phytic acid was determined using the phytic acid (total phosphorus) Megazyme assay (Megazyme, Wicklow, Ireland; catalog number: K-PHYT 05/17).

#### 2.2.2. Amino Acid Profile

The amino acid profile of the whole cakes and their fractions was analyzed by ion exchange chromatography using an Automatic Amino Acid Analyzer (Biochrom 30+, Biochrom, Cambridge, UK), following the method described by Tomičić et al. [27]. The analysis involved the separation of amino acids via strong cation exchange chromatography followed by a ninhydrin color reaction and photometric detection at 570 nm, except for proline, which was detected at 440 nm. Amino acids were identified by comparing the retention times with those of a standard amino acid solution (Sigma-Aldrich, St. Louis, MI, USA). Results were expressed as gram of amino acid per 100 g of sample.

#### 2.2.3. Fatty Acid Profile

Extraction of lipids from the samples was performed by cold extraction using a mixture of chloroform–methanol (2:1) for 2.5 h. The extracted lipids were converted to fatty acid methyl esters (FAME) and then injected into an Agilent 7890A gas chromatograph with a flame ionization detector (Agilent Technologies, Santa Clara, CA, USA). The separation was performed on a fused silica capillary column SP-2560 (100 m × 0.25 mm, d = 0.20 μm; Supelco, Bellefonte, PA, USA) under the conditions described in Ilić et al. [4]. The identification of FAME was performed using an authentic standard (Supelco 37 Component FAME Mix, Sigma-Aldrich, St. Louis, MI, USA), while the content was expressed in grams of each identified FAME per 100 g total FAME.

#### 2.2.4. Tocopherols

Determination of α-, β-, and γ-tocopherols was performed according to the standardized method [28], using high-performance liquid chromatography with fluorescence detection (HPLC-FLD) and utilizing an Agilent 1260 Infinity system (Agilent Technologies, Santa Clara, CA, USA). The system was equipped with a normal-phase column (Phenomenex Luna Silica, 5 µm, 250 mm × 4.6 mm). Detection was performed with an excitation wavelength of 290 nm and an emission wavelength of 330 nm. Results were expressed as milligrams of tocopherols per kilogram of dried sample.

#### 2.2.5. Antioxidative Potential

The ethanolic extracts from the samples were prepared using ultrasound-assisted extraction with 60% (*v*/*v*) ethanol, a liquid-to-solid ratio of 10 mL/g, and an extraction temperature of 60 °C for 45 min. An 8 L ultrasonic bath with a generator power of 360 W was used to facilitate the extraction of the desired compounds under reflux conditions. After extraction, the liquid extract was separated from the plant material by vacuum filtration. The concentration of the obtained extracts was determined by drying a 3 mL aliquot at 105 °C in a laboratory oven until constant weight. The remaining liquid was stored in a refrigerator.

The total polyphenolic content (TPC) in the ethanolic extracts was determined according to the previously described spectrophotometric method [29]. The absorbance of the sample was monitored at 765 nm after an incubation period of 90 min using a Varian Cary 100 spectrophotometer (Mulgrave, Victoria, Australia). The content was expressed as gallic acid equivalent per 100 g of dry weight.

The antioxidative potential of camelina cake was determined using the DPPH assay reported by Savić Gajić et al. [30]. The mixture was incubated for 30 min, and the absorbance was measured at 517 nm. The antioxidant potential was determined by calculating the half-maximal inhibitory concentration (IC_50_), interpolated from the dose–response curve of DPPH radical inhibition versus sample concentration.

#### 2.2.6. Color Measurements

Color measurements were carried out using the Chroma Meter color analyzer (Model CR-400, Minolta Co., Osaka, Japan) with attachment CR-A33f. The results were expressed according to the CIELab color system, where the color values were expressed as L* (lightness/brightness), a* (redness/greenness), and b* (yellowness/blueness).

#### 2.2.7. Water and Oil Absorption Capacity

The determination of water absorption capacity (WAC) and oil absorption capacity (OAC) was performed following the method described by Kain et al. [31]. The results for WAC are presented as g of water per g of sample, while the results for OAC are presented as gram of oil per gram of sample.

### 2.3. Statistical Analysis

The data are presented as mean ± standard deviation. All experimental runs were carried out in triplicate. The software Statistica 14 [32] was used for the data analysis. The effect of fractionation on the examined parameters was analyzed individually within each camelina variety. Data were subjected to the one-way analysis of variance (ANOVA), and the means were compared using the Fisher test. Differences were considered to be significant at a probability level of *p* < 0.05. Pearson correlation was used in the statistical analysis to evaluate the linear relationship between TPC and the IC_50_ values obtained from the DPPH assay.

## 3. Results

### 3.1. Yield of the Camelina Cake Fractions

**Figure 2:**
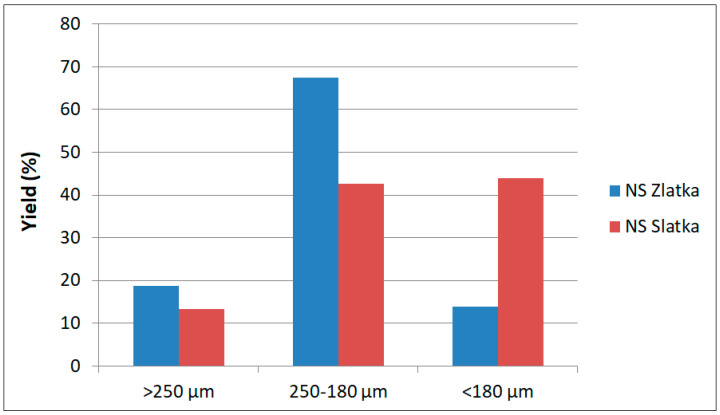
Yield of the camelina seed cake fractions.

The yields of the sieving fractions are presented in Table 1. After sieving the milled camelina cake, three particle size fractions were obtained: >250 μm, 250–180 μm, and <180 μm. The fraction yields, calculated relative to the initial whole camelina cake, are shown in Figure 2. As can be seen, the distribution of fractions differed between the tested camelina varieties. The coarsest fraction (>250 μm), which was primarily composed of hulls, accounted for 18.7% in the NS Zlatka variety and 13.3% in the NS Slatka variety. The largest differences were observed in the medium (250–180 μm) and fine (<180 μm) fractions, consisting mainly of cotyledon particles. The medium fraction yield was 67.5% for NS Zlatka and 42.7% for NS Slatka, while the fine fraction yield was 13.8% for NS Zlatka and 44.0% for NS Slatka.

### 3.2. Proximate Composition of Camelina Cake Fractions

Table 1 presents the proximate composition of camelina cakes and their fractions obtained after sieving.

Across all samples, protein content ranged from 29.8% to 40.3%, fat content from 11.9% to 19.4%, and ash content from 4.7% to 6.3%. The sieving led to an increase in both crude protein and fat content as particle size decreased, regardless of camelina variety. Crude fiber content ranged from 5.9% to 15.3% and linearly increased with increasing cake particle sizes in both the NS Zlatka and NS Slatka varieties. Glucosinolates were found in substantial concentrations in camelina seed cake and its fractions, and their levels were observed to increase with decreasing particle size in both camelina varieties studied. In NS Zlatka variety, glucosinolate concentrations ranged from 19.8 to 24.3 μmol/g, while NS Slatka exhibited higher values, ranging from 21.1 to 27.4 μmol/g. Generally, the NS Slatka variety demonstrated greater glucosinolate content in both the whole cake and its fractions than the NS Zlatka variety. The content of condensed tannins in camelina cakes and their fractions ranged from 0.5 to 2.2 g/kg. The lowest levels were observed in the finest fractions of both evaluated varieties (0.5 g/kg for NS Zlatka and 0.9 g/kg for NS Slatka). In both varieties, phytic acid levels increased as particle size in fractions decreased. In the NS Zlatka variety, the content ranged from 17.6 g/kg in the coarsest fraction to 41.1 g/kg in the finest fraction. In the NS Slatka variety, the content of phytic acid in the coarsest fraction was 20.6 g/kg, while in the finest fraction it was 38.4 g/kg. In both varieties, the phytic acid level in the medium-sized fraction (250–180 μm) was similar to that of the whole cake.

### 3.3. Amino Acid Content of Camelina Cake Fractions

The amino acid profiles of camelina cake and its fractions are presented in Table 2. A total of 17 amino acids were identified, with total amino acid content ranging from 27.86 to 37.42 g/100 g across the samples. Of this, the essential amino acids (EAA) content ranged from 12.28 to 16.89 g/100 g. Arginine was the most abundant essential amino acid, followed by leucine. Other EAAs, including isoleucine, lysine, threonine, phenylalanine, histidine, and valine, were present in lower concentrations. Similarly to the distribution of crude protein, the finest fractions exhibited the highest EAA content, whereas the coarsest fractions contained the lowest levels. Among the non-essential amino acids (NEAA), glutamic acid was the most abundant, followed by aspartic acid, proline, and serine. Total NEAA content ranged from 15.69 to 20.44 g/100 g. In general, the content of individual amino acids was higher in the finer fractions (<180 μm) and lowest in the coarser fractions (>250 μm).

### 3.4. Fatty Acid Content of Camelina Seed Fractions

Camelina seed cake and its fractions were characterized by a low content of saturated fatty acids (SFA) (10.4–11.4%), among which the most prevalent was palmitic acid (C16:0), followed by stearic acid (C18:0) (Table 3).

The medium-sized fraction (250–180 μm) exhibited the highest total SFA content, primarily due to its elevated level of palmitic acid. The content of monounsaturated fatty acids (MUFA) in camelina cake and its fractions was between 31.2 and 33.3%. In the NS Zlatka variety, the fractionation of camelina cake did not significantly (*p* > 0.05) affect total MUFA content. In contrast, the finest fraction of the NS Slatka variety had the highest MUFA content, including the greatest level of oleic acid (C18:1n9). In the NS Zlatka variety, however, the highest oleic acid level was observed in the medium-sized fraction. The content of gondoic acid (C20:1n9) ranged between 13.7 and 14.8%. In the NS Zlatka variety, the content of this fatty acid remained consistent across all fractions, whereas in the NS Slatka variety, the whole cake exhibited the highest level of gondoic acid. PUFA represented the most abundant group of fatty acids in camelina cake and its fractions (55.6–58.8%). The predominant PUFA was α-linolenic acid (C18:3n3, ALA), present in amounts ranging from 32.4% to 35.4%. In the NS Zlatka variety, the highest ALA amount was observed in the whole cake, whereas in NS Slatka, it was most abundant in the coarsest fraction. Linoleic acid (C18:2n6, LA) was the second most abundant PUFA, with the highest content found in the medium-sized fraction. Additionally, erucic acid (C22:1n9) was present in all cake fractions, with levels ranging from 2.5% in the medium-sized fraction to 3.3% in the whole cake and coarsest fraction. A noteworthy feature of camelina cake and its fractions was their high content of omega-3 PUFA, ranging between 33.7% and 36.8%. The content of omega-6 PUFA ranged from 21.4% to 22.6%. The omega-6 to omega-3 ratio was low, between 0.6 and 0.7.

### 3.5. Tocopherol Content of Camelina Cake Fractions

The concentrations of tocopherols in the camelina cake fractions are presented in Table 4. Both α-tocopherol and β-tocopherol were below the limit of detection. The concentration of γ-tocopherol in the fractions increased as particle size decreased, with the finest fraction exhibiting the highest γ-tocopherol content. In the NS Zlatka variety, γ-tocopherol levels ranged from 196.4 to 266.0 mg/kg, representing a 40% increase from the coarsest to the finest fraction. In the NS Slatka variety, the γ-tocopherol content ranged from 126.1 mg/kg in the coarsest fraction to 254.3 mg/kg in the finest fraction, indicating a twofold increase with fractionation.

### 3.6. Total Polyphenolic Content of Camelina Cake Fractions

The TPC in camelina seed cake fractions ranged between 0.51 and 0.58 g GAE/100 g d.m. (Table 5). Statistically significant differences (*p* < 0.05) were observed only between the whole cake and the coarsest fraction in the NS Zlatka variety.

### 3.7. Antioxidant Potential of Camelina Cake Fractions and Correlation Analysis

The antioxidant potential of camelina cake fractions was evaluated based on their IC_50_ values, revealing statistically significant differences (*p* < 0.05) between the fractions for both varieties (Table 5). For the NS Zlatka variety, the lowest IC_50_ value, indicating the highest antiradical activity, was observed in the whole cake. In contrast, for the NS Slatka variety, the highest antioxidant potential (i.e., the lowest IC_50_ value) was found in the fractions > 250 µm.

Correlation analysis between the IC_50_ values and both the TPC and γ-tocopherol content of the camelina cake fractions was performed to assess the main contributors to antiradical activity. Regarding TPC, the coefficients of determination (R^2^) were 0.381 and 0.670 for the NS Zlatka and NS Slatka varieties, respectively. This suggests a moderate positive correlation between polyphenol content and antiradical activity, especially in the NS Slatka variety. The relationship between the IC_50_ values and γ-tocopherol content was evaluated, resulting in R^2^ values of 0.463 for NS Zlatka and 0.077 for the NS Slatka variety.

### 3.8. Color Properties of Camelina Cake Fractions

The color parameters (CIE L*, a*, and b* coordinates) of camelina cake fractions are presented in Table 6. As can be seen, all measured color scores were significantly (*p* < 0.05) affected by the particle size fractionation in both evaluated camelina varieties. The lightness (L*) of the camelina cake fractions increased with decreasing particle size and varied between 50.9 for the coarsest fraction to 78.1 for the finest fraction. The highest value of red color (a*) had the coarsest fraction (10.6 for NS Zlatka variety and 10.3 for NS Slatka variety), and it decreased with decreasing particle size. A very low value of a* was observed for the finest fraction of camelina cake from the NS Slatka variety. A negative and low value of the color parameter a* was observed in the finest fraction of camelina cake from the NS Zlatka variety. In contrast to redness, the yellowness (b*) of the fractions increased as particle size decreased, with the finest fraction exhibiting the most intense yellow color. The values of b* ranged between 26.2 for the coarsest fraction to 31.3 for the finest fraction.

### 3.9. Water and Oil Absorption Properties of Camelina Cake Fractions

The water absorption capacity (WAC) and oil absorption capacity (OAC) of camelina cake and its fractions are presented in Table 7. For the NS Zlatka variety, WAC values ranged from 3.62 to 9.31 g/g, while for the NS Slatka variety, they ranged from 5.55 to 9.61 g/g. In both varieties, the highest WAC was observed in the coarsest fraction, with values gradually decreasing as particle size decreased. In the NS Zlatka variety, the medium-sized fraction exhibited the highest OAC, while the finest fraction showed the lowest OAC. In contrast, no significant differences (*p* > 0.05) in OAC were observed among the camelina cake fractions of NS Slatka variety.

## 4. Discussion

### 4.1. Effect of Sieving on Proximate Composition

The contents of protein, fat, and ash were consistent with those reported in previous studies [2,33]. The protein content of camelina cake was comparable to that of other oilseed cakes such as rapeseed and linseed, which are commonly used as protein sources in animal feed [34]. However, it remained lower than that of soybean meal, a more widely used protein-rich ingredient. The fat and ash contents of camelina cake were in accordance with those reported for hempseed, rapeseed, sunflower, and pumpkin cakes [34]. Sieving caused a concentration of protein and fat in the finest fractions (<180 µm). Comparable observations regarding the enrichment of protein and fat in finer particle fractions of other oilseed cakes have been documented by Hansen et al. [35] for rapeseed meal, Pojić et al. [17] for hempseed cake, and Bárta et al. [18] for flax, milk thistle, and sunflower cakes. Owing to its rich protein content, camelina cake can serve as a valuable ingredient in animal nutrition. Additionally, a product with such high protein content, particularly in the fine fractions, holds potential for various food applications aimed at enhancing nutritional value. These protein-rich fractions could be incorporated into meat analogs or reformulated meat products, such as meatballs and patties, where part of the meat could be replaced with camelina-derived protein to improve nutritional profiles and support more sustainable food production. The high residual fat content observed in the whole cake, and particularly in the finest fraction, presents both opportunities and limitations in its application as a protein-rich ingredient. From a functional standpoint, the elevated fat levels can be advantageous in food formulations, as they may allow for the partial substitution of animal-derived fats such as butter. This substitution not only improves the nutritional profile by reducing saturated fat content but also aligns with consumer demand for more sustainable and plant-based ingredients. However, the presence of high fat content, particularly from camelina oil, which is rich in PUFA, also introduces notable challenges. PUFA are highly susceptible to oxidative degradation, which can negatively affect the shelf-life, sensory properties, and overall stability of the final product. It was proposed that a maximum fat content of 25% in oilseed cake flours would be produced to maintain oxidative stability and ensure compatibility with other formulation components [36]. In the context of animal feed production, high fat levels can act as a lubricant during pelleting, reducing friction between particles and the pellet die wall, which may negatively affect the overall quality of the pellets. Nevertheless, these challenges can be effectively managed through appropriate adjustments in feed formulation and processing parameters. The content of crude fiber in the observed cake was in agreement with those reported by others [11,37]. In general, the crude fiber content of camelina cake was comparable to that of rapeseed and linseed cakes, but lower than that of hempseed, sunflower, and pumpkin cakes [34]. The highest content of crude fiber was found in the coarsest fraction (>250 μm), as expected, as fibers are mainly found in the outer layers of the seed. Previous studies have demonstrated that the coarse fraction contains higher fiber levels and a greater proportion of hulls compared to the finer fraction, which is consistent with the findings of the present study [35]. Crude fibers are indispensable in ruminant nutrition because they support microbial fermentation, positively impact ruminant performance, and enhance milk yield and fat content [38]. Chuang et al. [39] encouraged the use of high-fiber agricultural by-products as animal feed and feed additives, given their multiple benefits. In food products, the incorporation of the camelina cake fraction with the highest fiber content would also be considered beneficial due to the positive impact of fibers on gastrointestinal function. Consequently, the coarsest fraction of camelina seed cake, owing to its high fiber content, may be utilized as a partial flour substitute in the formulation of bread products [40], as well as in crackers or high-protein, high-fiber biscuits.

Similar concentrations of glucosinolates in camelina cake have been reported in previous studies, consistent with the findings of the present study [37,41,42]. Sieving resulted in an increased concentration of glucosinolates as the particle size of the fractions decreased. Similarly, Pojić et al. [17] reported a higher concentration of glucosinolates in finer particle size fractions of hempseed cake. In the context of animal nutrition, they are considered among the most significant antinutritional factors present in camelina cake. Although camelina cake contains more glucosinolates than rapeseed meal, there are structural differences between the glucosinolate profiles of the two. The dominant glucosinolate in camelina is glucocamelinin (10-methylsulfinyldecyl glucosinolate), accounting for approximately 62–72% of the total, while glucoarabin (9-methylsulfinylnonyl) and 11-methylsulfinylundecyl glucosinolates represent roughly 30% and 10%, respectively. These compounds are considered to be less harmful than progoitrin (found in rapeseed), sinigrin (found in Brasica carinata) and glucoiberin (present in broccoli), due to their lower reactivity and reduced conversion into toxic metabolites like goitrin [43]. Nevertheless, elevated glucosinolate content remains a major limitation to the broader use of camelina cake in animal feeds. While glucosinolates have long been regarded as antinutritional factors, emerging evidence suggests that, at low concentrations, they may also offer health-promoting effects. Their hydrolysis products, particularly isothiocyanates, have demonstrated antioxidant and anti-inflammatory properties and have been shown to prevent cardiovascular diseases, carcinogenesis, tumor growth, and metastasis in human studies [44]. These findings suggest that, when present in controlled amounts, glucosinolates from camelina could provide functional benefits in human food and animal diets as well.

The content of condensed tannins in the camelina cakes and their fractions was similar to the levels reported in previous research [42,45,46]. Condensed tannins, also known as proanthocyanidins, are primarily concentrated in the seed hulls of oilseeds and legumes. During milling and subsequent sieving, hull particles tend to accumulate in coarser fractions, while finer fractions are enriched in cotyledon material, which contains a smaller amount of tannins. Although the coarsest fraction (>250 μm) was expected to concentrate tannins due to the presence of hulls, its condensed tannin content was comparable to that of the whole seed cake. In contrast, the finest fraction, being predominantly cotyledon-derived, showed the lowest condensed tannin content. Condensed tannins can reduce digestion in both monogastric animals and ruminants. However, these negative impacts are typically observed only when condensed tannins are present at levels exceeding 1% of the diet [45]. In the current study, the concentration of condensed tannins in camelina cakes and fractions was found to be relatively low, suggesting that their inclusion in animal feed is unlikely to pose any significant nutritional risk. On the contrary, when present in small amounts, condensed tannins may offer health-promoting properties [45]. Tannins have been applied in various sectors, including as dietary supplements, nutraceuticals, functional food additives, and pharmaceutical agents. Products containing tannins are widely recognized for their diverse biological activities, which include antioxidant, anticancer, antidiabetic, antiallergic, antimutagenic, antiaging, and antimicrobial properties [47].

The content of condensed tannins in the camelina cakes and their fractions was similar to the levels reported in previous research [42,45,46]. Condensed tannins, also known as proanthocyanidins, are primarily concentrated in the seed hulls of oilseeds and legumes. During milling and subsequent sieving, hull particles tend to accumulate in coarser fractions, while finer fractions are enriched in cotyledon material, which contains a smaller amount of tannins. Although the coarsest fraction (>250 μm) was expected to concentrate tannins due to the presence of hulls, its condensed tannin content was comparable to that of the whole seed cake. In contrast, the finest fraction, being predominantly cotyledon-derived, showed the lowest condensed tannin content. Condensed tannins can reduce digestion in both monogastric animals and ruminants. However, these negative impacts are typically observed only when condensed tannins are present at levels exceeding 1% of the diet [45]. In the current study, the concentration of condensed tannins in camelina cakes and fractions was found to be relatively low, suggesting that their inclusion in animal feed is unlikely to pose any significant nutritional risk. On the contrary, when present in small amounts, condensed tannins may offer health-promoting properties [45]. Tannins have been applied in various sectors, including as dietary supplements, nutraceuticals, functional food additives, and pharmaceutical agents. Products containing tannins are widely recognized for their diverse biological activities, which include antioxidant, anticancer, antidiabetic, antiallergic, antimutagenic, antiaging, and antimicrobial properties [47].

The levels of phytic acid observed in camelina cakes and their fractions are consistent with findings from previous studies [45,48,49]. Increasing concentration of phytic acid in camelina cake fractions with decreasing particle size was in accordance with Pojić et al. [17]. Phytic acid is recognized for its ability to bind phosphorus, making it unavailable for absorption. This poses a nutritional challenge for monogastric animals, which lack the enzyme phytase, which is needed to hydrolyze phytate-bound phosphorus. Interestingly, recent research has also highlighted the potential health benefits of phytic acid, particularly its antioxidant, antimicrobial, and anticancer properties [49]. Therefore, while high levels of phytic acid may reduce nutrient availability in monogastric diets, its presence in moderate amounts may also contribute to animal health when balanced appropriately in dietary formulations.

### 4.2. Effect of Sieving on Amino Acid Composition

The amino acid profile observed in this study aligns with findings reported by Almeida et al. [41] and Kim et al. [50]. The amino acid composition, particularly the profile of EAAs, is a critical factor in determining the biological value of a protein source. Among EAAs, lysine and methionine are frequently the most limiting in many plant-based dietary protein sources. Notably, the lysine content in camelina seed cake and its fractions was higher than in hempseed and linseed cakes, and comparable to that of rapeseed cake [51]. Methionine content in camelina cake was higher than in linseed and rapeseed cakes but lower than in hempseed cake, with the exception of the finest fraction of NS Slatka, which had a methionine content similar to that of hempseed cake [51]. Overall, the amino acid composition of camelina cake is comparable to that of rapeseed meal, highlighting its potential as a high-quality protein source. Similarly to the content of protein, the finest fractions had the highest EAA, NEAA, and total amino acid content, whereas the coarsest fractions had the lowest levels. Given its high crude protein content and favorable essential amino acid profile, camelina cake and its fractions represent a promising protein ingredient for both animal feed and human nutrition applications.

### 4.3. Effect of Sieving on Fatty Acid Composition

Camelina cake possesses a distinctive fatty acid profile, characterized by a low content of SFA and an exceptionally high proportion of unsaturated fatty acids, which account for approximately 90% of its total fatty acid content. The SFA content in camelina cake and its fractions was comparable to that reported in hempseed and linseed cake and lower than in pumpkin seed cake, but higher than in sunflower seed and rapeseed cake [34]. Camelina cake exhibits a higher MUFA content compared to hempseed cake yet this remains considerably lower than the levels reported in rapeseed cake, pumpkin seed cake, and soybean meal [34,52]. Camelina cake contains a significant amount of gondoic acid, which has been reported to exhibit anti-inflammatory and hepatoprotective effects, support insulin secretion, reduce lipid accumulation, and potentially contribute to neurodevelopment [53,54]. A less abundant MUFA in camelina cake is erucic acid, present at approximately 3%. This fatty acid is considered harmful to both animals and humans, as high dietary intake has been associated with myocardial lipidosis, a condition marked by excessive fat accumulation in heart tissues, and other pathological cardiac lesions [55]. In the present study, the erucic acid content in camelina cake was found to be below the regulatory threshold set by European Commission (2006) [56]. The PUFA content in camelina seed cake was higher than that in rapeseed and sunflower seed cakes, comparable to that of pumpkin seed cake, but lower than in hempseed and linseed cakes [34]. The major PUFAs in camelina cake are LA and ALA, both of which are classified as essential fatty acids. ALA and LA, which belong to the omega-3 and omega-6 PUFA families, respectively, are crucial for maintaining cell membrane integrity, supporting brain development, regulating inflammation, contributing to the production of bioactive lipid mediators and supporting overall metabolic health [57]. Camelina cake contained higher ALA levels than the cakes of pumpkin seed, hempseed, and rapeseed and soybean meal, but lower than linseed cake [34,52]. Camelina cake and its fractions exhibited a low omega-6/omega-3 ratio (below 1), which is considered optimal for maintaining health. This ratio was more favorable than those observed in commonly used oilseed cakes such as rapeseed, hempseed, sunflower, and pumpkin seed cakes [34]. Increasing omega-3 intake and lowering the omega-6/omega-3 ratio have been associated with better management of rheumatoid arthritis and asthma, as well as potential protective effects against breast, prostate, colon, and kidney cancers [57]. These findings highlight the high nutritional value of camelina cake and its potential as a valuable ingredient in food and feed formulations, helping to enhance omega-3 intake and promote a healthier fatty acid balance in the diet. Furthermore, the inclusion of camelina cake in the diets of animals such as ruminants, laying hens, and broilers has proven to be an efficient way to produce animal-derived products, such as milk, eggs, and meat, with an increased content of omega-3 fatty acids [11,12,58,59].

### 4.4. Effect of Sieving on Tocopherol Content

The cold pressing of camelina seeds resulted in the majority of tocopherols being extracted into the oil fraction, particularly γ-tocopherol, with concentrations of 385.1 mg/kg in the NS Zlatka variety and 547.4 mg/kg in the NS Slatka variety, as reported in our previous study [60]. However, due to the presence of residual oil in the cold-pressed cake, γ-tocopherols were also detected in this by-product. The levels of γ-tocopherol in camelina cake were higher than those in cold-pressed rapeseed, sunflower seed, flaxseed and safflower cakes [61]. γ-tocopherol possessed notable biological activity, primarily attributed to its potent antioxidant, anti-inflammatory, and anticancer properties, making it a promising agent for preventing and treating oxidative stress-related diseases. It can modulate inflammatory pathways and has shown potential to inhibit tumor growth, induce apoptosis, and suppress angiogenesis, particularly in prostate, lung, and colon cancers [62]. Given the well-documented health benefits of γ-tocopherol, incorporating camelina cake, especially its finest fraction, which is rich in this compound, into human and animal diets may positively impact overall health. This potential health-promoting effect warrants further investigation, particularly in the context of animal nutrition strategies and functional food development. Furthermore, the use of camelina cake in food and feed formulations can enhance the antioxidant capacity of the diet and prolong product shelf life. This was demonstrated in a previous study by Orczewska-Dudek et al. [12], where supplementation of broiler diets with 10% camelina cake increased the γ-tocopherol content in the feed by 110%, resulting in greater accumulation in muscle cell membranes and subsequently reducing lipid oxidation in the breast muscle.

### 4.5. Effect of Sieving on Phenolic Content and Antioxidative Potential

Camelina cake and its fractions can be considered a major source of phenolic compounds [63]. This content is important for the estimation of the nutritive value of the cake and depends on numerous factors, including growing conditions [64], variety [65], extraction methods, and solvent used [66]. These factors can significantly affect the TPC, so their monitoring is key to achieving the desired extract properties. The TPC was close to that obtained for camelina seed cultivated in Poland [67], but significantly higher compared to the methanolic extract (0.137 mg GAE/mL) of camelina cultivated in Poland [66]. The extract of camelina cake can potentially be used as an initial raw material in the development of various functional products or for other purposes. Kramar et al. [63] found that the extract of camelina cake, containing 795.5 mg/L of polyphenols, efficiently inhibits biodiesel oxidation. Ethanolic extracts of camelina cake were classified as strong to moderate antioxidants depending on the analyzed fraction, according to the classification proposed by Molyneux et al. [68]. Based on IC_50_ values across both varieties, it can be concluded that their antiradical activities were nearly equivalent. The antiradical activity observed in the various fractions is likely due to the presence of polyphenolic compounds [69]. Numerous studies have shown that extracts from camelina cake possess strong antioxidant potential, as confirmed by various in vitro assays such as DPPH, ABTS, and FRAP [63]. Owing to their antioxidant properties, camelina cake extracts represent a promising natural alternative to synthetic antioxidants, offering a safer profile with fewer side effects. The higher R^2^ of γ-tocopherol in the NS Zlatka variety indicates that γ-tocopherol may play a more significant role in its antiradical activity compared to polyphenols. Conversely, the very low correlation in the NS Slatka variety suggests that γ-tocopherol contributes little to its antioxidant potential. These findings highlight distinct antioxidant mechanisms in the two varieties. In the NS Zlatka variety, γ-tocopherols appear to be the predominant contributors to antiradical activity, whereas in the NS Slatka variety, polyphenolic compounds are likely the primary antioxidants. This variety-dependent variation underscores the complexity of antiradical activity in camelina cake and suggests that multiple bioactive compounds act synergistically. Understanding these differences is crucial for optimizing the use of camelina cake extracts as natural antioxidants in food, feed and pharmaceutical applications.

### 4.6. Effect of Sieving on Color Properties

In food products intended for human consumption, color is a key quality attribute that significantly influences consumer perception and acceptance. Color characteristics can be connected to the abundance of biologically active components such as pigments or phenolic substances [18]. Finer camelina cake fractions have a larger specific surface area and scatter light more effectively, which can make fractions appear lighter. In contrast, coarser particles tend to absorb more light, resulting in a darker appearance of the cake fraction [17]. After oil extraction from camelina seeds, a press cake remains, retaining the majority of phenolic compounds from the seed [70]. Fractionation by sieving showed that the coarsest fraction contained the outer seed layers, where phenolic compounds are concentrated. The red coloration observed in coarser fractions is likely attributed to the presence of anthocyanins, a subclass of phenolic compounds naturally occurring in camelina [71]. The negative and low value of the color parameter a* in the finest fraction of camelina cake from the NS Zlatka variety indicates a slight greenish coloration, likely due to the presence of chlorophyll pigments, which are also present in camelina oil [72]. The yellow coloration observed in the whole press cake and its fractions can be attributed to the presence of carotenoid pigments, which may be desirable in various food applications. Although the oil is mechanically extracted from camelina seeds, the resulting press cake still retains a considerable amount of residual oil (approximately 15%). Camelina oil is naturally rich in carotenoids, including β-carotene, lutein, and zeaxanthine [72], which are responsible for its characteristic yellow-orange color.

### 4.7. Effect of Sieving on WAC and OAC

WAC is considered an important property of oilseed cakes intended for application in food and feed products. The exceptionally high WAC observed in the >250 µm fraction suggests that 1 g of this coarse material can absorb nearly ten times its own weight in water. This high water absorption capacity is likely due to the higher content of fibrous and structural components in the outer seed layers, which are more prevalent in the coarser fractions retained during sieving. Additionally, the presence of mucilage in camelina cake further contributes to its high WAC, as mucilage has strong water-binding properties and forms a gel-like matrix upon hydration. As reported by Fabre et al. [73], 7–10% of the camelina seed mass is composed of mucilage, which mainly consists of polysaccharides (∼80%) such as arabinose, galactose, glucose, rhamnose, and xylose, and co-existing proteoglycans. These compounds can cause a significant increase in viscosity in solutions, even at low concentrations, which is why they are widely used in both food and non-food industries as thickeners, swelling agents, foam stabilizers, gelling agents, emulsifiers, water-binding agents, etc. [74]. Cakes or flours with high water absorption capacity have proven to be valuable ingredients in soups and sauces, as well as in bakery products, as they enhance texture and moisture retention, thereby contributing to improved juiciness and extended freshness of the final products [75,76]. This property of is also highly desirable in the meat industry, where additives with strong water-binding capacity are often used to improve texture and yield. In contrast, when camelina cake is used as a feed ingredient, its mucilage content can adhere to the cake matrix, reducing its nutritional value and leading to slower fat assimilation in livestock [77]. The obtained results are consistent with those reported for flours from lentil [78]. In this study, camelina cake and their fractions showed low OAC. These lower OAC values suggest a reduced presence of hydrophobic proteins and limited lipid-binding capacity, resulting in decreased oil retention ability [79]. The ability of cake to absorb oil is an important parameter in the baking industry, as it reflects its emulsifying functionality [78].

## 5. Conclusions

This study demonstrates that fractionation significantly affects the nutritional and functional properties of cold-pressed camelina cake fractions. The finest fraction (<180 µm) obtained from both camelina varieties demonstrated the highest levels of protein, fat, essential amino acids, and γ-tocopherols, and the lowest concentration of condensed tannins. Visually, this fraction exhibited a lighter color with an enhanced yellow color. However, it also contained the highest concentrations of antinutritional compounds, namely glucosinolates and phytic acid. In contrast, the coarsest fraction (>250 µm) had an increased crude fiber content, higher antioxidant potential, the greatest water absorption capacity, and a darker color with a more pronounced redness. It also showed the lowest concentrations of glucosinolates and phytic acid. Camelina cake and its derived fractions offer significant potential as sustainable, multifunctional ingredients across various sectors. In animal nutrition, they serve as high-quality protein sources enriched with bioactive compounds. In human diets, camelina fractions can be strategically incorporated into a wide range of food products, particularly those tailored for vegan and vegetarian consumers, depending on the desired nutritional and functional effects. Fractions with higher levels of antinutritional compounds should be included at optimized levels to ensure safety. Additionally, these fractions present valuable opportunities as substrates for the extraction of bioactive compounds and may find further application in the cosmetic and pharmaceutical industries.

## Figures and Tables

**Table 1:** Proximate composition of camelina cake fractions.

Parameter	NS Zlatka				NS Slatka			
	Whole Cake	>250 µm	250–180 µm	<180 µm	Whole Cake	>250 µm	250–180 µm	<180 µm
Moisture, %	7.7 ± 0.04 ^b^	8.2 ± 0.07 ^c^	7.5 ± 0.01 ^b^	6.4 ± 0.12 ^a^	7.8± 0.06 ^b^	8.4 ± 0.11 ^d^	8.1 ± 0.04 ^c^	7.1 ± 0.01 ^a^
Protein, %	35.1 ± 0.02 ^b^	33.6 ± 0.07 ^a^	36.3 ± 0.01 ^c^	39.4 ± 0.08 ^d^	36.3 ± 0.15 ^c^	29.8 ± 0.06 ^a^	34.0 ± 0.01 ^b^	40.3 ± 0.11 ^d^
Fat, %	16.2 ± 0.04 ^b^	12.8 ± 0.11 ^a^	16.1 ± 0.34 ^b^	19.4 ± 0.01 ^c^	14.8 ± 0.06 ^c^	11.9 ± 0.17 ^a^	13.4 ± 0.14 ^b^	17.7 ± 0.08 ^d^
Ash, %	4.8 ± 0.02 ^a^	4.7 ± 0.01 ^a^	6.3 ± 1.16 ^a^	5.0 ± 0.04 ^a^	4.8 ± 0.06 ^a^	4.7 ± 0.05 ^a^	4.8 ± 0.00 ^a^	5.1 ± 0.08 ^b^
Crude fiber, %	9.3 ± 0.21 ^b^	13.0 ± 0.59 ^c^	10.1 ± 0.89 ^b^	5.9 ± 0.52 ^a^	8.8 ± 0.09 ^b^	15.3 ± 0.03 ^d^	11.0 ± 0.31 ^c^	6.8 ± 0.67 ^a^
Glucosinolates, μmol/g	21.9 ± 0.27 ^b^	19.8 ± 0.13 ^a^	21.3 ± 0.01 ^b^	24.3 ± 0.34 ^c^	24.9 ± 0.23 ^b^	21.1 ± 0.26 ^a^	24.2 ± 0.61 ^b^	27.4 ± 0.19 ^c^
Condensed tannins, g/kg	1.8 ± 0.02 ^b^	1.5 ± 0.06 ^b^	1.5 ± 0.40 ^b^	0.5 ± 0.13 ^a^	2.2 ± 0.07 ^b^	1.4 ± 0.55 ^ab^	2.1 ± 0.17 ^b^	0.9 ± 0.03 ^a^
Phytic acid, g/kg	30.7 ± 0.06 ^b^	17.6 ± 2.32 ^a^	31.1 ± 0.72 ^b^	41.1 ± 3.47 ^c^	33.1 ± 0.45 ^b^	20.6 ± 0.63 ^a^	33.6 ± 0.70 ^b^	38.4 ± 1.13 ^c^

Values within each variety were analyzed separately, and different letters in rows denote statistically significant differences at *p* < 0.05.

**Table 2:** Amino acid profile of camelina cake fractions.

Amino Acids (g/100 g Sample)	NS Zlatka				NS Slatka			
	Whole Cake	>250 µm	250–180 µm	<180 µm	Whole Cake	>250 µm	250–180 µm	<180 µm
Leucine	2.34 ± 0.02 ^ab^	2.28 ± 0.09 ^a^	2.49 ± 0.06 ^b^	2.76 ± 0.08 ^c^	2.39 ± 0.03 ^c^	1.84 ± 0.05 ^a^	2.13 ± 0.06 ^b^	2.75 ± 0.06 ^d^
Isoleucine	1.36 ± 0.05 ^a^	1.27 ± 0.03 ^a^	1.53 ± 0.07 ^b^	1.62 ± 0.03 ^b^	1.47 ± 0.01 ^b^	1.18 ± 0.07 ^a^	1.25 ± 0.06 ^a^	1.64 ± 0.05 ^c^
Lysine	1.56 ± 0.04 ^a^	1.54 ± 0.05 ^a^	1.63 ± 0.06 ^ab^	1.75 ± 0.07 ^b^	1.62 ± 0.04 ^b^	1.43 ± 0.06 ^a^	1.54 ± 0.05 ^ab^	1.77 ± 0.05 ^c^
Methionine	0.65 ± 0.05 ^b^	0.43 ± 0.04 ^a^	0.57 ± 0.04 ^b^	0.64 ± 0.04 ^b^	0.65 ± 0.04 ^b^	0.34 ± 0.04 ^a^	0.66 ± 0.04 ^b^	0.73 ± 0.05 ^c^
Threonine	1.33 ± 0.05 ^ab^	1.28 ± 0.03 ^a^	1.35 ± 0.06 ^ab^	1.45 ± 0.04 ^b^	1.40 ± 0.01 ^ab^	1.27 ± 0.07 ^a^	1.35 ± 0.07 ^ab^	1.43 ± 0.06 ^c^
Phenylalanine	1.92 ± 0.05 ^b^	1.74 ± 0.07 ^a^	2.05 ± 0.05 ^b^	2.24 ± 0.08 ^c^	2.08 ± 0.03 ^c^	1.59 ± 0.06 ^a^	1.77 ± 0.06 ^b^	2.23 ± 0.03 ^d^
Histidine	1.07 ± 0.03 ^ab^	0.97 ± 0.06 ^a^	1.15 ± 0.04 ^bc^	1.20 ± 0.04 ^c^	1.16 ± 0.04 ^b^	0.85 ± 0.04 ^a^	0.940.06 ^a^	1.31 ± 0.05 ^c^
Arginine	3.09 ± 0.06 ^b^	2.84 ± 0.08 ^a^	3.13 ± 0.06 ^b^	3.52 ± 0.03 ^c^	3.23 ± 0.02 ^c^	2.52 ± 0.03 ^a^	2.93 ± 0.08 ^b^	3.51 ± 0.04 ^d^
Valine	1.51 ± 0.02 ^ab^	1.48 ± 0.03 ^a^	1.58 ± 0.03 ^b^	1.70 ± 0.05 ^c^	1.53 ± 0.03 ^b^	1.27 ± 0.02 ^a^	1.49 ± 0.05 ^b^	1.70 ± 0.03 ^c^
EAA	14.83 ± 0.05 ^b^	13.83 ± 0.28 ^a^	15.50 ± 0.01 ^c^	16.89 ± 0.09 ^d^	15.54 ± 0.03 ^c^	12.28 ± 0.03 ^a^	14.06 ± 0.07 ^b^	17.08 ± 0.10 ^d^
Alanine	1.29 ± 0.06 ^a^	1.28 ± 0.05 ^a^	1.37 ± 0.06 ^ab^	1.48 ± 0.03 ^b^	1.35 ± 0.03 ^b^	1.18 ± 0.02 ^a^	1.34 ± 0.05 ^b^	1.42 ± 0.04 ^b^
Aspartic acid	3.74 ± 0.04 ^ab^	3.66 ± 0.04 ^a^	3.86 ± 0.07 ^b^	4.22 ± 0.07 ^c^	3.67 ± 0.04 ^c^	3.26 ± 0.04 ^a^	3.54 ± 0.05 ^b^	4.14 ± 0.05 ^d^
Glutamic acid	5.70 ± 0.05 ^b^	5.51 ± 0.08 ^a^	5.75 ± 0.05 ^b^	6.16 ± 0.06 ^c^	5.33 ± 0.06 ^b^	4.69 ± 0.05 ^a^	5.41 ± 0.06 ^b^	6.14 ± 0.05 ^c^
Glycine	1.85 ± 0.20 ^a^	1.66 ± 0.06 ^a^	1.78 ± 0.03 ^a^	1.89 ± 0.05 ^a^	1.65 ± 0.04 ^ab^	1.51 ± 0.03 ^a^	1.69 ± 0.05 ^bc^	1.82 ± 0.07 ^c^
Cystine	n.d	n.d	n.d	n.d	n.d	n.d	n.d	n.d
Proline	2.46 ± 0.05 ^b^	2.13 ± 0.05 ^a^	2.52 ± 0.03 ^b^	2.84 ± 0.03 ^c^	2.62 ± 0.08 ^c^	2.01 ± 0.05 ^a^	2.34 ± 0.06 ^b^	2.95 ± 0.05 ^d^
Serine	2.17 ± 0.03 ^a^	2.14 ± 0.05 ^a^	2.25 ± 0.06 ^bc^	2.35 ± 0.08 ^c^	2.17 ± 0.04 ^b^	1.95 ± 0.05 ^a^	2.06 ± 0.06 ^ab^	2.30 ± 0.06 ^c^
Tyrosine	1.39 ± 0.02 ^ab^	1.35 ± 0.06 ^a^	1.42 ± 0.05 ^ab^	1.49 ± 0.06 ^b^	1.41 ± 0.10 ^b^	1.07 ± 0.09 ^a^	1.14 ± 0.06 ^a^	1.58 ± 0.06 ^b^
NEAA	18.59 ± 0.11 ^b^	17.73 ± 0.08 ^a^	18.96 ± 0.25 ^b^	20.44 ± 0.20 ^c^	18.24 ± 0.24 ^c^	15.69 ± 0.15 ^a^	17.49 ± 0.07 ^b^	19.86 ± 0.25 ^d^
TAA	33.42 ± 0.16 ^b^	31.56 ± 0.20 ^a^	34.46 ± 0.26 ^c^	37.33 ± 0.10 ^d^	33.88 ± 0.08 ^c^	27.86 ± 0.13 ^a^	31.72 ± 0.06 ^b^	37.42 ± 0.01 ^d^

EAA—essential amino acids; NEAA—non-essential amino acids; TAA—total amino acids. Values within each variety were analyzed separately, and different letters in rows denote statistically significant differences at *p* < 0.05.

**Table 3:** Fatty acid profile of camelina cake fractions.

Fatty Acid (g/100 g)	NS Zlatka				NS Slatka			
	Whole Cake	>250 µm	250–180 µm	<180 µm	Whole Cake	>250 µm	250–180 µm	<180 µm
C14:0	0.1 ± 0.01	0.1 ± 0.01	0.1 ± 0.00	0.1 ± 0.00	0.1 ± 0.01	0.1 ± 0.02	0.1 ± 0.00	0.1 ± 0.00
C16:0	6.6 ± 0.01 ^a^	6.8 ± 0.00 ^b^	7.1 ± 0.04 ^c^	6.9 ± 0.07 ^b^	6.4 ± 0.02 ^a^	6.4 ± 0.04 ^a^	7.4 ± 0.01 ^b^	7.2 ± 0.22 ^b^
C18:0	2.7 ± 0.02	2.7 ± 0.02	2.7 ± 0.03	2.7 ± 0.04	2.5 ± 0.01	2.5 ± 0.04	2.4 ± 0.02	2.6 ± 0.02
C20:0	1.6 ± 0.03	1.6 ± 0.05	1.5 ± 0.03	1.6 ± 0.02	1.4 ± 0.04 ^ab^	1.5 ± 0.03 ^b^	1.3 ± 0.01 ^ab^	1.2 ± 0.01 ^a^
SFA	11.0 ± 0.00 ^a^	11.2 ± 0.04 ^b^	11.4 ± 0.04 ^c^	11.3 ± 0.08 ^bc^	10.4 ± 0.02 ^a^	10.4 ± 0.03 ^a^	11.2 ± 0.03 ^b^	11.1 ± 0.23 ^b^
C16:1	0.2 ± 0.05	0.2 ± 0.06	0.2 ± 0.06	0.1 ± 0.00	0.2 ± 0.05	0.2 ± 0.05	0.2 ± 0.07	0.2 ± 0.04
C18:1 n9 c	13.8 ± 0.03 ^a^	13.8 ± 0.06 ^a^	14.9 ± 0.04 ^c^	14.3 ± 0.09 ^b^	13.8 ± 0.08 ^b^	13.6 ± 0.04 ^a^	15.1 ± 0.02 ^c^	15.9 ± 0.12 ^d^
C20:1 n9	14.0 ± 0.77	14.1 ± 0.05	13.7 ± 0.46	14.4 ± 0.62	14.8 ± 0.02 ^c^	13.9 ± 0.50 ^ab^	13.6 ± 0.17 ^a^	14.6 ± 0.08 ^bc^
MUFA	31.2 ± 0.69	31.3 ± 0.13	31.7 ± 0.38	32.0 ± 0.48	31.8 ± 0.06 ^b^	30.8 ± 0.54 ^a^	31.4 ± 0.19 ^ab^	33.3 ± 0.00 ^c^
C18:2 n6 c	19.3 ± 0.01 ^a^	19.9 ± 0.06 ^b^	20.1 ± 0.05 ^c^	19.3 ± 0.09 ^a^	19.4 ± 0.14 ^a^	19.9 ± 0.07 ^b^	20.7 ± 0.03 ^d^	20.2 ± 0.11 ^c^
C18:3 n3	35.0 ± 0.81 ^b^	34.0 ± 0.41 ^ab^	33.4 ± 0.39 ^a^	33.9 ± 0.39 ^ab^	34.9 ± 0.35 ^c^	35.4 ± 0.50 ^c^	33.6 ± 0.27 ^b^	32.4 ± 0.17 ^a^
C20:2 n6	2.1 ± 0.00	2.1 ± 0.07	2.0 ± 0.00	2.0 ± 0.07	2.0 ± 0.03 ^b^	2.1 ± 0.05 ^b^	1.8 ± 0.02 ^a^	1.8 ± 0.12 ^a^
C22:1 n9	3.3 ± 0.00 ^b^	3.3 ± 0.09 ^b^	3.0 ± 0.03 ^a^	3.2 ± 0.05 ^b^	3.0 ± 0.01 ^bc^	3.1 ± 0.04 ^c^	2.5 ± 0.07 ^a^	2.7 ± 0.24 ^ab^
C20:3 n3	1.5 ± 0.13	1.5 ± 0.10	1.4 ± 0.09	1.5 ± 0.08	1.4 ± 0.10	1.4 ± 0.10	1.3 ± 0.01	1.3 ± 0.16
PUFA	57.8 ± 0.70	57.5 ± 0.18	56.9 ± 0.34	56.7 ± 0.40	57.7 ± 0.08 ^b^	58.8 ± 0.58 ^c^	57.4 ± 0.22 ^b^	55.6 ± 0.24 ^a^
ω-3 PUFA	36.5 ± 0.68 ^b^	35.5 ± 0.31 ^ab^	34.8 ± 0.30 ^a^	35.4 ± 0.31 ^ab^	36.3 ± 0.25 ^c^	36.8 ± 0.60 ^c^	34.8 ± 0.22 ^b^	33.7 ± 0.24 ^a^
ω-6 PUFA	21.4 ± 0.01 ^a^	22.0 ± 0.13 ^b^	22.1 ± 0.04 ^b^	21.4 ± 0.09 ^a^	21.4 ± 0.17 ^a^	22.0 ± 0.02 ^b^	22.6 ± 0.04 ^c^	22.0 ± 0.24 ^b^
ω-6/ω-3	0.6 ± 0.01	0.6 ± 0.01	0.6 ± 0.00	0.6 ± 0.00	0.6 ± 0.01 ^a^	0.6 ± 0.01 ^a^	0.7 ± 0.01 ^b^	0.7 ± 0.01 ^b^

SFA—saturated fatty acids; MUFA—monounsaturated fatty acids; PUFA—polyunsaturated fatty acids. Values within each variety were analyzed separately, and different letters in rows denote statistically significant differences at *p* < 0.05.

**Table 4:** Content of tocopherols in camelina cake fractions.

Parameter, mg/kg	NS Zlatka				NS Slatka			
	Whole Cake	>250 µm	250–180 µm	<180 µm	Whole Cake	>250 µm	250–180 µm	<180 µm
α-tocopherol	<0.1	<0.1	<0.1	<0.1	<0.1	<0.1	<0.1	<0.1
β-tocopherol	<0.1	<0.1	<0.1	<0.1	<0.1	<0.1	<0.1	<0.1
γ-tocopherol	188.6 ± 1.84 ^a^	196.4 ± 3.54 ^a^	233.2 ± 4.95 ^b^	266.0 ± 12.30 ^c^	162.3 ± 11.10 ^b^	126.1 ± 8.13 ^a^	218.5 ± 8.06 ^c^	254.3 ± 5.80 ^d^

Values within each variety were analyzed separately, and different letters in rows denote statistically significant differences at *p* < 0.05.

**Table 5:** Total polyphenolic content (TPC) and antioxidative potential of camelina cake fractions.

Parameter	NS Zlatka				NS Slatka			
	Whole Cake	>250 µm	250–180 µm	<180 µm	Whole Cake	>250 µm	250–180 µm	<180 µm
TPC (g GAE/100 g)	0.58 ± 0.03 ^b^	0.52 ± 0.02 ^a^	0.55 ± 0.02 ^ab^	0.55 ± 0.04 ^ab^	0.51 ± 0.02 ^a^	0.53 ± 0.02 ^a^	0.51 ± 0.03 ^a^	0.52 ± 0.02 ^a^
IC_50_ (μg/mL)	80.3 ± 2.40 ^a^	108.2 ± 3.24 ^b^	91.9 ± 2.76 ^c^	122.4 ± 3.66 ^d^	123.4 ± 3.70 ^c^	83.4 ± 2.50 ^b^	101.9 ± 3.06 ^a^	104.9 ± 3.15 ^a^

Values within each variety were analyzed separately, and different letters in rows denote statistically significant differences at *p* < 0.05.

**Table 6:** Color of camelina cake fractions.

Parameter	NS Zlatka				NS Slatka			
	Whole Cake	>250 µm	250–180 µm	<180 µm	Whole Cake	>250 µm	250–180 µm	<180 µm
L*	70.5 ± 0.03 ^c^	50.9 ± 0.03 ^a^	69.0 ± 0.02 ^b^	78.1 ± 0.02 ^d^	69.9 ± 0.01 ^c^	51.2 ± 0.01 ^a^	61.9 ± 0.01 ^b^	76.8 ± 0.02 ^d^
a*	2.6 ± 0.03 ^b^	10.6 ± 0.01 ^d^	3.6 ± 0.02 ^c^	−0.1 ± 0.02 ^a^	3.1 ± 0.02 ^b^	10.3 ± 0.02 ^d^	6.7 ± 0.03 ^c^	0.8 ± 0.02 ^a^
b*	28.4 ± 0.02 ^b^	26.7 ± 0.02 ^a^	29.5 ± 0.02 ^c^	31.0 ± 0.02 ^d^	28.2 ± 0.02 ^a^	27.5 ± 0.01 ^c^	28.6 ± 0.02 ^b^	31.3 ± 0.01 ^d^

Values within each variety were analyzed separately, and different letters in rows denote statistically significant differences at *p* < 0.05.

**Table 7:** Water and oil absorption characteristics of camelina cake fractions.

Parameter	NS Zlatka				NS Slatka			
	Whole Cake	>250 µm	250–180 µm	<180 µm	Whole Cake	>250 µm	250–180 µm	<180 µm
WAC (g/g)	9.31 ^c^	9.55 ^c^	8.48 ^b^	3.62 ^a^	8.85 ^b^	9.61 ^c^	8.36 ^b^	5.55 ^a^
OAC (g/g)	1.18 ^b^	1.34 ^b^	1.40 ^c^	0.99 ^a^	1.69 ^a^	1.48 ^a^	1.58 ^a^	1.64 ^a^

Values within each variety were analyzed separately, and different letters in rows denote statistically significant differences at *p* < 0.05.

## Data Availability

The original contributions presented in this study are included in the article. Further inquiries can be directed to the corresponding author.

## Abbreviations

The following abbreviations are used in this manuscript:

FAME	fatty acid methyl esters
SFA	saturated fatty acids
MUFA	monounsaturated fatty acids
PUFA	polyunsaturated fatty acids
LA	linoleic acid
ALA	α-linolenic acid
EAA	essential amino acids
NEAA	non-essential amino acids
TAA	total amino acids
TPC	total polyphenolic content
IC50	half-maximal inhibitory concentration
WAC	water absorption capacity
OAC	oil absorption capacity
